# Lexical and Cognitive Underpinnings of Verbal Fluency: Evidence from Bengali-English Bilingual Aphasia

**DOI:** 10.3390/bs10100155

**Published:** 2020-10-08

**Authors:** Abhijeet Patra, Arpita Bose, Theodoros Marinis

**Affiliations:** 1 School of Psychology and Clinical Language Sciences, University of Reading, Reading RG6 7BE, UK; t.marinis@uni-konstanz.de; 2 Moss Rehabilitation Research Institute, Elkins Park, PA 19027, USA; 3 Department of Linguistics, University of Konstanz, D-78457 Konstanz, Germany

**Keywords:** aphasia, bengali, bilingual, executive control, inhibition, cluster, switches, semantic fluency, letter fluency, timing

## Abstract

Research in bilingual healthy controls (BHC) has illustrated that detailed characterization of verbal fluency along with separate measures of executive control stand to inform our understanding of the lexical and cognitive underpinnings of the task. Such data are currently lacking in bilinguals with aphasia (BWA). We aimed to compare the characteristics of verbal fluency performance (semantic, letter) in Bengali–English BWA and BHC, in terms of cross-linguistic differences, variation on the parameters of bilingualism, and cognitive underpinnings. BWA showed significant differences on verbal fluency variables where executive control demands were higher (fluency difference score, number of switches, between-cluster pauses); whilst performed similarly on variables where executive control demands were lower (cluster size, within-cluster pauses). Despite clear cross-linguistic advantage in Bengali for BHC, no cross-linguistic differences were noted in BWA. BWA who were most affected in the independent executive control measures also showed greater impairment in letter fluency condition. Correlation analyses revealed a significant relationship for BWA between inhibitory control and number of correct responses, initial retrieval time, and number of switches. This research contributes to the debate of underlying mechanisms of word retrieval deficits in aphasia, and adds to the nascent literature of BWA in South Asian languages.

## 1. Introduction

More than half of the world population is bi/multilingual [1]. There has been a surge in research in bi/multilingual populations in the past decade. A quick search with the term “bilingualism” on PubMed provides 8761 results (1946–2020) and 65% (5759 results) of these studies were published since 2010. Bilingual speakers juggle between two sets of languages in their day-to-day conversation, and previous studies have shown that bilingualism influences linguistic, cognitive, and brain functioning in healthy adults [2]. One of the central issues in the bilingual psycholinguistic research is to understand the relationship between word retrieval, executive control, and cross-linguistic lexical activation. As the bilingual population grows worldwide, clinical population involving bilingual speakers also increases. Therefore, it is critical to understand the relationship between these processes (lexical and executive control) in the bilingual clinical population to improve assessment and treatment approaches [3]. Verbal fluency tasks—quick and easy to administer and a part of routine neuropsychological assessment protocol—have been extensively used to examine such relationships between executive control and language functioning in healthy adults [4,5,6], and in clinical populations [7,8,9,10,11]. However, there are only a handful of studies that have studied the word retrieval difficulties and its relationship to executive control processes using verbal fluency tasks in bilinguals with aphasia [12,13,14,15,16]. This issue is more pertinent especially in the context of South Asian languages, as they are under-reported in the literature and there is a strong bias towards Germanic and Romance languages in aphasia research [17]. 

In this research, we sought to examine the relationship between word retrieval, executive control, and cross-linguistic differences in a group of Bengali–English bilinguals with aphasia (BWA) and bilingual healthy controls (BHC) using verbal fluency tasks. First, we examined the differences in word retrieval abilities between BWA and BHC to understand the extent of word retrieval impairment in BWA. Second, to understand how varying executive control demand within the verbal fluency tasks can impact performance, we characterized verbal fluency performance using a wide range of variables where linguistic and executive control demands are differential (See Table 1). For example, the ability to switch between subcategories and duration of between-cluster pauses have been attributed to the greater executive control demand of the task [7,18,19]; whilst cluster size and duration of within-cluster pauses have been attributed to the greater lexical control demand of the task [20,21]. Table 1 provides a description of these variables and provides a relative classification based on whether a specific verbal fluency variable requires greater executive control or lexical control processing. Third, we administered three independent measures of executive control processes, namely the Stroop task (measuring selective inhibition, [22,23]), the Trail Making Test (TMT, measuring shifting between mental sets, [24]), and the backward digital span (measuring verbal working memory, [25]) to further examine the relationship between word retrieval and different executive control processes. Fourth, to explore cross-linguistic differences we administered the verbal fluency tasks in both languages (Bengali and English) and carefully characterized our participants on various bilingualism related variables (such as age of language of acquisition, language of instruction, language proficiency, language usage, and dominance). This multi-pronged approach enabled us to specify the involvement of executive control and cross-linguistic lexical activation in verbal fluency performance in BWA.

Typically, verbal fluency tasks have two conditions: Semantic and letter. In the semantic condition, also referred to as category fluency, participants are asked to generate as many unique words as possible from a given semantic category (e.g., animals). In the letter condition, participants are asked to produce as many unique words as possible that start with a given letter (e.g., F) or phoneme (/p/). In the semantic fluency condition, participants are required to revisit the existing links in their mental lexicon related to a category while producing words: For example, when participants are asked to produce words from animal category, and a participant names dog, all related animal nodes in the mental lexicon that share features with dog get activated [26]. However, such activation becomes detrimental in the letter fluency condition where participants need to produce words with a specific letter/phoneme and require suppression of the activation of related semantic concepts [27,28]. 

Research comparing semantic and letter fluency conditions have shown fundamental differences between these two. Most studies have argued in favor of greater demand on executive control in the letter fluency condition [6,19,27,28,29,30,31,32,33], some studies have argued against such a notion [34,35]. For example, in a recent study [6], we found vocabulary-matched healthy bilinguals outperformed monolinguals in the letter fluency condition, which could be attributed to the bilingual advantage as observed from the separate executive control measures (specifically inhibitory control and mental-set shifting). In a cross-sectional study, Gordon and colleagues [34] investigated the effect of ageing on verbal fluency tasks and found monolingual older adults were more affected in semantic fluency compared to letter fluency condition. The authors attributed the age-related protection in the letter fluency condition to vocabulary knowledge. However, Ljungberg and colleagues [36] did not find such ageing effect in bilingual healthy controls. On a similar line, Friesen and colleagues [27] found letter fluency performance to be impacted by bilingual status and not by age. It is noteworthy to mention that Gordon and colleagues did observe digit span (measuring verbal working memory) playing a greater role in letter fluency compared to semantic fluency, and suggested that greater goal maintenance (i.e., inhibition of semantic clusters) is necessary to perform in letter fluency. In summary, these differences in lexical and executive control demands between the two conditions make verbal fluency task a suitable experimental paradigm to examine the relationship between executive control and language functioning in BWA population. 

Another way of examining the role of executive control in verbal fluency performance is to move beyond the traditional approach of calculating the number of correct responses [28,33]. In Table 1, we have outlined these variables. Specifically, studies have shown that executive control demands are higher in some verbal fluency variables (e.g., fluency difference score, [6,27]; switching from one subcategory to another, [19]) compared to others (e.g., searching words within a subcategory; [6,10]). For example, the ability to switch often and quickly between once subcategory to another has been linked to executive control abilities [7,18,19]. Similarly, Friesen and colleagues [27] have argued that a smaller difference score between the two conditions as a proportion of the semantic fluency score (i.e., fluency difference score) is related to superior executive control abilities in healthy controls. 

An important variable in the verbal fluency literature is the mean retrieval latency (also called Sub-RT) that is the time-point where half of the responses have been produced. Friesen and colleagues [27] have linked longer Sub-RT in conjunction with fewer numbers of correct responses to greater cross-linguistic interference in healthy BHC. Rohrer et al. [38] have linked faster Sub-RT in conjunction with fewer number of correct responses to the structural loss in the mental lexicon in individuals with Alzheimer’s disease. Using such extensive approach of analysis in BHC, we have been able to establish that going beyond number of CR and semantic versus letter fluency dissociation, is crucial for understanding the involvement of lexical and executive control processes in verbal fluency tasks [6]. However, such detailed investigation is lacking in the BWA literature and the present study aims to fill a significant gap by extensively characterizing the verbal fluency performance in BWA. 

In contrast to the appreciable verbal fluency literature in bilingual healthy controls, verbal fluency studies in BWA are limited. Using a semantic fluency task, Kiran et al. [15] have found that Spanish-English BWA produced fewer words and fewer switches than healthy controls. However, both groups employed similar clustering strategy. Crucially, unlike healthy controls, BWA did not demonstrate cross-linguistic differences at the group level. Better performance for healthy controls in their dominant language is consistent with the hypothesis that performing the task in the non-dominant language require greater cognitive effort, resulting in lower output [39]. However, BWA showed parallel impairment and/or recovery pattern. No cross-linguistic differences have also been shown in a recent study with bilingual individuals with traumatic brain injury using verbal fluency task [11]. Similarly, Roberts and Le Dorze [16] investigated French–English BWA using semantic fluency and did not find cross-linguistic differences in the number of correct responses and in the error pattern. Previous studies in BWA have investigated the cross-linguistic differences in bilingual groups where both languages are structurally similar (e.g., Spanish–English, French–English). This research adds to the literature by examining cross-linguistic performance in BWA where languages are linguistically different and belonging to different families (Bengali: Part of the Indo-Aryan language family, and English: Part of the Indo-European language family). South Asian languages, such as Bengali, are under-reported and under-explored in current aphasia research. 

Faroqi-Shah et al. [13] investigated the relationship between word retrieval and executive control in BWA using semantic fluency, picture naming, and Stroop tasks. The authors found no correlation between the semantic fluency and the Stroop task, but observed a strong correlation between picture naming and the semantic fluency condition. The lack of a correlation between inhibitory control and semantic fluency was attributed to impaired executive control abilities in aphasia that are no longer available to support word retrieval or possibly that the Stroop task may not be an appropriate task to tap into the executive control mechanisms that underpin semantic fluency performance. Based on Miyake et al.’s [40] influential work on executive control, we measured three components of executive control—inhibitory control (verbal Stroop test), mental set-shifting (TMT), and working memory (Backward digit span test)—to better understand which component (or components) of executive control taps into the executive control mechanisms underlying verbal fluency tasks. We decided to use a combination of experimental (verbal Stroop) and clinical (TMT and backward digit span) executive control measures. The choice of the task was primarily based on the feasibility of using them with the neurological populations (e.g., aphasia), and availability of literature on these tasks for comparisons.

Recent work by Carpenter et al. [12] found BWA to be more sensitive to the effects of increased executive control demand on a verbal fluency task relative to healthy controls. Participants (13 Spanish-English BWA and 22 BHC) performed the tasks in four conditions for the semantic fluency: No-Switch in L1 (NS-L1), No-Switch in L2 (NS-L2), Self-Switch (SS), and Forced Switch (FS). The authors hypothesized that the SS condition (can respond in any language) would require the lowest cognitive effort, whereas the FS (required to switch from one language to another within task) and NS condition (standard verbal fluency task where instruction is given prior to the task in which language participants have to produce words) would place greater demand on the executive control mechanism. Results revealed no significant difference between the group for the easier SS condition, but BWA group produced a significantly lower number of correct responses in the FS and NS condition. Results from this study demonstrated that the BWA group was sensitive to the higher executive control demand in the verbal fluency tasks. In terms of cross-linguistic difference, BWA did not show cross-linguistic difference, but healthy controls showed cross-linguistic difference in letter fluency. The authors advocated that the lack of cross-linguistic difference for BWA in the difficult letter fluency condition suggestive of the fact that increased executive control demand in letter fluency may have hindered performance in the BWA group. 

### 1.1. The Current Investigation, Research Questions, and Predictions

Although the above studies have made a significant advancement in our understanding of the relationship between executive control and verbal fluency performance in BWA, several issues remain unresolved. We aim to address these issues in the present study and discuss how this research advances our understanding of word production in BWA using verbal fluency task. 

1) The above-mentioned studies have only investigated semantic fluency (except the study by Carpenter and colleagues [12]), although previous research has shown a greater involvement of executive control abilities in letter fluency performance [6,12] (see [34] for a contrasting view). Given that executive control differences have been attributed to the performance advantage in verbal fluency in healthy controls [6,28], our understanding of verbal fluency in aphasia will benefit from a systematic comparison of semantic and letter fluency at a group and individual level. In the present study, we have compared verbal fluency performance between eight Bengali–English BWA and eight BHC by including both semantic (Bengali and English: Animals, fruits and vegetables) and letter (English: F, A, S; Bengali: P, K, M) fluency conditions. We analyzed the verbal fluency data both at the group level as well as at the individual level. Given that heterogeneity in performance is a hallmark feature of any aphasia group studies, we provide individual level analyses for standardly reported verbal fluency variables (number of correct responses, FDS, cluster sizes, number of switches) and executive control measures. This approach provides a depth to our understanding of aphasia performance, which is often missed if we rely solely on group means. 

2) Most studies on verbal fluency in aphasia have restricted their analysis to only the number of correct responses, with very few studies venturing into clustering and switching analyses. Previous studies with healthy controls have shown that extensive characterization of verbal fluency performance is crucial for the comprehensive understanding of the interaction of executive control and lexical processes [6]. This has not yet been attempted with BWA; the present research will be a step towards changing in our understanding of word production and executive control in BWA. We characterized verbal fluency performance in terms of quantitative (number of correct responses, fluency proportion difference score), time-course (1st-RT, Sub-RT), as well as clustering and switching (cluster size, number of switches, within-cluster pauses, between-cluster pauses) measures (see Table 1 for a complete description of these variables).

3) There is a lack of studies addressing the relationship between verbal fluency and executive control in BWA. To the best of our knowledge, Faroqi-Shah et al.’s [13] study was the only study which attempted to link verbal fluency performance with separate executive control measures in BWA. However, the authors measured only the number of correct responses for the semantic fluency task and inhibitory control for the executive control measure. To characterize the executive control abilities of our participants, we employed three independent measures of executive control (inhibitory control, mental-set shifting, and working memory) and characterized the verbal fluency using different variables (see Table 1). 

4) Most studies in aphasia have investigated speakers of Germanic and Romance languages (96.29% of all the articles), especially English [17]. The most under represented languages in the aphasia research are Arabic, Hindi, Portuguese, Russian, and Bengali. In the present study, we have reported data from an under-explored South Asian language (Bengali) and population (Bengali–English bilingual speakers). We provided detailed characterization of our participants on relevant variables for bilingualism: Language history and acquisition patterns, usage patterns, proficiency, and dominance. In addition, we characterized our BWA on type, severity of aphasia, as well as their post-stroke linguistic profile (naming, repetition, word-to-picture matching, and reading aloud) in both languages. Our research fills a significant gap in the aphasia literature by researching and reporting on Bengali–English bilingual speakers. Bengali (also known as Bangla) is an Indo-European language spoken in South Asia by people from Bangladesh, Eastern states of India, and a significant Bengali diaspora. Bengali is currently ranked as the seventh most spoken language in the world, and worldwide more than 265 million people speak Bengali as their first or second language in their day to day life. Bengali is the national language of Bangladesh (first language of 142 million speakers, 98.8% of the total population, Bangladesh Census, 2011) and the official language of three states of India—West Bengal, Tripura, and Assam (first language of 97 million speakers, 8.3% of the total population, India Census, 2011). Despite the large number of Bengali speakers, only a handful of studies have reported on epidemiological aspects of aphasia research in Bengali speakers, rather than linguistic investigation per se [41,42].

The study had the following specific research aims and predictions:

1. To determine differences in verbal fluency performance (quantitative, time-course, as well as clustering and switching analysis) between BWA and BHC.

We predict the BWA group to produce fewer correct responses in both semantic and letter fluency as compared to BHC. However, based on the existing literature [6,7,12], we expect BWA to have greater difficulty where executive control demand is higher (e.g., letter fluency, FDS, number of switches, between-cluster pauses). For the BWA individual analysis, we expect a similar pattern that is greater difficulty in the letter fluency condition and for the variables where executive control demand is higher. In terms of the cross-linguistic comparison, similarly to Kiran et al.’s study [15], at group level we expect no cross-linguistic differences for the BWA in contrast to the BHC. However, the present study group involves speakers from a different language background (Bengali), and we aim to explore if there is any cross-linguistic difference due to differences in the language family.

2. To establish the relationship between verbal fluency performance and executive control abilities for BWA and BHC.

Based on previous research [6], we expect that executive control measures (especially, inhibitory control) may correlate significantly with high executive control demanding verbal fluency measures (e.g., FDS, number of switches, between-cluster pauses) for both BWA and BHC, but the strength of the correlation should be different in the two groups. Specifically, we expect BWA to have a stronger correlation between executive control and verbal fluency measures compared to BHC. This prediction is an extrapolation from the observation that low proficient bilinguals engage with their executive control mechanism while performing a task in their less proficient language [43]. Following on the same rationale, BWA would need to recruit their executive control processes more while performing on the verbal fluency task to compensate for their lexical difficulties. However, we acknowledge that correlational analysis based on a sample size of eight could be considered underpowered and interpretation needs to be approached with caution (see [44] for a review). To mitigate these concerns (at least false positive) from the correlation analysis, we have set our significance level at *p* < 0.01. We would like to highlight the fact that a small sample size such as this is not unusual in clinical studies, especially where participants belong from an under-represented group in the literature. 

## 2. Methods

### 2.1. Participants

Eight Bengali–English BWA (*M* = 47.75 years, *SD* = 11.9) and eight Bengali–English BHC (*M* = 43.13 years, *SD* = 15.30) participated in this study. Participants were recruited via contacts with certified speech-language therapists from Kolkata, India. Participation in this study was voluntary, and participants provided written consent prior to participation. All the procedures were approved by the University of Reading Research Ethics Committee (Ethical approval code: 2014/060/AB).

All BWA had sustained a single left hemisphere cerebrovascular accident (CVA) resulting in aphasia at least six months prior to participation. Medical and neurological reports were reviewed to establish the participants’ medical history. All participants were right-handed (pre-stroke for BWA) and had at least twelve years of education (BWA: *M* = 16.63 years, *SD* = 2.33; BHC: *M* = 16.88 years, *SD* = 1.88). There was no history of other neurological conditions, alcohol or drug abuse, neuropsychiatric conditions or dementia. There was no significant difference between BWA and BHC on age, sex, and years of education (all *p*s > 0.05, see Table 2). The demographic and neurological details of the BWA are summarized in Table 2. We administered the WAB-R in English [45] and its adapted version in Bengali [46] to assess the type and severity of aphasia in both languages. Individual performances on the WAB-R (both languages) are provided in Appendix A (Table A1). Based on WAB, all BWA showed good auditory comprehension, but demonstrated variable levels of difficulty in spoken language production, naming, and repetition. As can be seen from Table 2, the BWA group presented with non-fluent aphasia with mild to moderate severity in both languages, except for BWA6, who had severe aphasia in English and was not available for testing in Bengali. 

As there is no comprehensive psycholinguistic test, such as the Psycholinguistic Assessments of Language Processing in Aphasia [47], which is culturally and linguistically appropriate in Bengali, we chose to administer the test battery developed by Croft and colleagues [48]. This battery measures single word production and comprehension in each language (Bengali and English). The battery included picture naming, spoken word-to-picture matching, word repetition, and reading aloud tasks. The same 30 nouns were included for the different tasks, which allowed comparison of performance across tasks. Individual level performance in this test battery is provided in the Appendix A (Table A2). In summary, the following pattern emerges from the Croft’s test battery: Intact spoken word comprehension; as a group BWA had difficulties in picture naming with semantic errors in the target language and translation equivalent cross-linguistic errors; relatively preserved repetition with formal errors but no cross-linguistic errors; and relatively preserved reading aloud abilities. Therefore, we can assume that our BWA showed an intact semantic system with deficits either in the phonological output lexicon or in lexical access (accessing the phonological word form from the semantic system).

### 2.2. Background Measures

#### 2.2.1. Bilingualism measures 

All speakers completed a set of subjective language background questionnaires (language acquisition history, language of instruction, self-rated language proficiency, language usage, and language dominance). To measure language acquisition history, instruction of language during education, self-rated language proficiency (in speaking, comprehension, reading, and writing), and the current language usage pattern, we adapted and modified the questionnaire developed by Muñoz et al. [49]. Language dominance was measured using the language dominance questionnaire [50]. Interested readers can access the adapted versions of these questionnaires in Patra et al. [6].

BWA speakers completed the self-rated language proficiency and language usage questionnaires twice to separately report their pre-stroke and post-stroke language abilities, with the support from caregiver or family members as needed. Language background scores obtained from the language background questionnaire are summarized in Table 3. Based on the questionnaires, there were no significant differences between the two groups (i.e., BHC vs. pre-stroke ratings for BWA) on the following variables: Language acquisition history, language of instruction during education, self-rated language proficiency, language usage, and language dominance (all *p* > 0.05, see Table 3). All participants were sequential bilinguals, that is, they had acquired Bengali before English (age of onset for English is 5 years or more). English was the language of instruction during higher education for all the participants. 

#### 2.2.2. Executive Control Measures 

Inhibitory control (Stroop test): The computerized Stroop Task used in this study was adapted from Scott and Wilshire [22]. Participants were assessed on two conditions, neutral and incongruent. In the neutral condition, participants named a series of 50 color rectangles, and in the incongruent condition a series of color words were presented with a different font color (e.g., RED word in green font color). Participants were asked to name the font color (e.g., green) of the color word (e.g., RED). Reaction Times (RT) were measured for the correct trials to calculate the Stroop difference (Equation (1)) and the Stroop ratio (Equation (2)). The Stroop ratio (Equation (2)) was used as a dependent variable to account for the overall slower response speed in the BWA compared to the BHC [6,13]. A smaller Stroop difference and Stroop ratio indicates better inhibitory control. Please refer to Patra et al. [6] for a detailed description of the task and analysis procedure.

(1) $$\mathrm{Stroop}\;\mathrm{difference}\;=\;{RT}_{INCONGRUENT\;TRIAL}-{RT}_{NEUTRAL\;TRIAL}$$

(2) $$\mathrm{Stroop}\;\mathrm{ratio}\;=\;\left[\frac{{RT}_{INCONGRUENT\;TRIAL}\;-\;{RT}_{NEUTRAL\;TRIAL}}{\frac{{RT}_{INCONGRUENT\;TRIAL}+\;{RT}_{NEUTRAL\;TRIAL}}{2}}\right]\times 100$$

Shifting between task-sets (Trail Making Test): The Trail Making Test (TMT, [24]), one of the most widely used neuropsychological tests, was used to assess mental set shifting [51]. The test consists of two parts, A and B. On part A, participants are asked to connect 25 circled numbers (e.g., 1, 2, 3, 4, etc.) distributed on a paper using a pen/pencil. On part B, participants need to connect the circles, but alternating between circled numbers and letters (e.g., 1, A, 2, B, 3, C, etc.). All participants completed both parts of the test. We measured the total time in seconds for both parts of the test, therefore achieving two scores, TMT-A and TMT-B. The dependent variables were: The TMT difference score (B-A), which has been shown to be the best indicator of task switching ability of the TMT test [51], and the TMT ratio (B/A), which has shown to control perceptual speed to some extent [52]. 

Working memory (Backward digit span test): Working memory was assessed using the backward digit span test from Wechsler Memory Scale (WMS 3, [25]). In this test, participants were verbally presented an increasingly longer series of digits from two to nine with a rate of presentation of one digit per second. Participants were asked to repeat the sequence of the digits in reverse order. The test ended when the participants failed on two consecutive trials at any one span size or when the maximum trial size was reached. The backward digit score was the total number of lists reported correctly in the backward digit span test. 

Statistical analysis and results: Independent samples t-test was conducted on the Stroop ratio measure, and non-parametric versions of independent samples t-test (Mann-Whitney U test) were used separately (as the data was not normally distributed) on the Stroop difference, TMT difference, TMT ratio, and the backward digit span measures between the groups. Table 4 shows that the two groups differed significantly only on the inhibitory control (Stroop ratio, Stroop difference), and mental-set shifting measures (TMT difference score, TMT ratio). Compared to BWA, BHC demonstrated a smaller Stroop difference and Stroop ratio, indicative of better inhibitory control, and a smaller TMT difference and TMT ratio, suggesting superior shifting ability. We did not find any difference on the working memory measure between the two groups. 

For the individual analysis, we performed Crawford and Howell’s [53] statistical method to compare each BWA’s score with the BHC group, and the results are provided in Table 5. Similar to the group results, we did not find any significant difference for the backward digit span variable (working memory). Individual variation was observed for inhibitory control and mental-set shifting variables. It is important to note that not all BWA had deficits in executive control. For inhibitory control (Stroop difference and Stroop ratio), BWA4, BWA5, and BWA7 performed similarly to BHC. For the mental shifting measure (TMT difference), BWA1, BWA4, and BWA7 performed similarly to the control, but when overall slowness was accounted for (TMT ratio), only BWA2 and BWA8 showed significantly worse performance than BHC. Overall, BWA2 (4 out of 5 variables), BWA3 (3 out of 5 variables), BWA6 (3 out of 5 variables), and BWA8 (4 out of 5 variables) were most affected across the variables when compared to BHC. This variability in performance at the individual level shows that having difficulty in one executive control component does not imply impairment across multiple domains, and executive control impairment at a group level does not reveal the complete picture (some individuals performed similarly to controls). Overall, the results show the complexity of studying aphasia. 

### 2.3. Verbal Fluency Measures

#### 2.3.1. Trials and Procedures

All participants completed two verbal fluency conditions—semantic and letter—in both languages. Participants never performed the task in both languages on the same day, and order of language was counterbalanced across participants. The conditions were counter-balanced across participants, that is, half of the participants performed the semantic fluency condition first and the other half performed the letter fluency condition first. After familiarizing with the task, participants were asked to produce as many words as possible in 60 s when the tester said “start”. The “start” prompt provided a definitive starting point for each trial. In the semantic fluency condition, participants produced words in two categories—animals, and fruits and vegetables. In the letter fluency condition, participants were asked to produce words that start with the letters F, A, and S for the English language and letters P, K, and M for the Bengali. The instruction for the Bengali letter fluency task was different from the English letter fluency task due to the phonology of Bengali language. In the Bengali letter fluency task, participants were asked to name words that start with the sound (e.g., /p/) rather than the letter (e.g., P). 

#### 2.3.2. Data Coding and Analysis

Responses were recorded with a digital voice recorder and all responses (including repetition and errors) were transcribed verbatim. Each correct response was time-stamped using PRAAT [55]. We measured the following variables for each trial:

- Number of correct responses (CR): Total number of responses produced in one-minute after excluding the errors. In semantic fluency, errors were repetition of same words, words from different semantic category (e.g., camel as a response for fruits and vegetables), and words from non-target language. In letter fluency, errors were repetition of same words, words beginning with a different letter (e.g., potato as a response for letter A), proper nouns (e.g., Australia as a response for letter A), same word with different endings (e.g., fast, faster, fastest were counted as single CR), and words from a non-target language.
- Fluency Difference Score (FDS): FDS was calculated by subtracting the CR in letter fluency from CR in semantic fluency and dividing the remainder with CR in semantic fluency (Equation (3)).
  (3) FDS= (CR semantic fluency−CR letter fluency)/CR semantic fluency
- First-RT (1st RT) and Subsequent-RT (Sub-RT): 1st RT is the time interval from the onset of the trial to the onset of the first response. 1st RT has been linked to the preparation time required to begin a task [38]. Sub-RT is the mean value of the time intervals from the 1st RT to the onset of each subsequent response. As mentioned earlier in the Introduction, Sub-RT provide estimation of mean retrieval latency and is associated with the declining rate of recall. A faster Sub-RT in conjunction with fewer CR indicates structural loss to the mental lexicon [38].
- Clustering and Switching: We derived four parameters to characterize the clustering and switching abilities of our participants—cluster size, number of switches, within-cluster pauses, between-cluster pauses. Following Troyer et al. [19], words that shared the same semantic subcategory constituted the semantic fluency cluster. Letter fluency cluster was determined when any one of these following criteria was met: Words that begin with same first two letters (fan and fat), words that differ only by a vowel sound (son and sun), rhyming words (stool and school), and homonyms (fair—legitimate, fare—money one has to pay in a public transport). Cluster size was calculated beginning with the second word in each cluster. Cluster size of zero was given for a single word (e.g., cat), cluster size of one was given for two words cluster (e.g., cat, dog belong to pet animal cluster and cluster size of one), and so on. Number of switches was the number of transitions between clusters. For example, cat, dog; leopard, cheetah; donkey, pig contain two switches—before leopard and after cheetah. For a detail description of clustering and switching analysis refer to Patra et al. [6].
- Within-cluster pause: Within-cluster pause was the mean time difference between successive words within a cluster. For example, cat, dog is a pet cluster, and onset time of cat is 3 sec and onset time of dog is 4 sec. Within-cluster pause for this cluster will be one second (i.e., 4 -3). A three-word cluster example can be pig, cow, horse, and with the onset time for pig, cow, and horse 5, 7, and 8, respectively. Within-cluster pause for this farm animal cluster will be ({(7 − 5) + (8 − 7)} / 2 = 1.5 sec).
- Between-cluster pauses: Between-cluster pauses refer to the time difference between the onset time of the last word of a cluster and first word of the consecutive cluster. An example of two consecutive clusters are cat, dog, and pig, cow, horse. The pause time between these clusters will be the difference between the onset time of dog and pig that is (5 − 4) = 1 sec.

## 3. Statistical Analysis

All variables were measured for each trial for the two fluency conditions for each participant in each language. To arrive at the mean score for each variable in each language, two trials were averaged for the semantic fluency, and three trials were averaged for the letter fluency condition. A three-way repeated measures ANOVA was used on the following variables: Number of CR, FDS, 1st-RT, sub-RT, cluster size, number of switches, within-cluster pause, and between-cluster pause. In the design, Group (BWA, BHC) was treated as a between-subject factor, while Language (Bengali, English) and Condition (Semantic, Letter; except for FDS) were treated as within-subject factors. Tukey’s post hoc tests were applied for significant interaction effects at *p* ≤ 0.05. 

To better understand the individual level variation and to capture the heterogeneity of BWA data, we compared each BWA’s score to the average score obtained from the control sample (BHC) for number of CR, FDS, cluster size, and number of switches [53,54], separately for semantic and letter fluency conditions (except for FDS). This comparison allowed us to examine the effect of condition at individual BWA level. Finally, to examine the relationship between the independent executive control measures (Stroop ratio, TMT ratio, and backward digit span) and the fluency variables, Spearman’s correlations were performed separately for each group, and significance value was set at *p* < 0.01.

## 4. Results

The mean and standard deviation values for the verbal fluency variables for Group (BWA; BHC), Language (Bengali; English), and Condition (Semantic; Letter) averaged across participants are presented in Table 6 (standard deviation reflects between-subject variation). The summary results of the statistical tests are also provided in Table 6 (See Table A3 in the Appendix A for statistical values). Statistical results from the individual level analysis are presented in Table 7. Findings from the correlation analyses between the executive control measures and verbal fluency variables for each group are presented in Table A4 in the Appendix A. The findings for Group differences are presented first, followed by the individual level performance. The findings on the relationship between the executive control measures and the verbal fluency variables are presented at the end. Item level raw data with time stamping is available from the University of Reading Research Data Archive [56].

### 4.1. Group Differences in Verbal Fluency Performance 

**Figure 1:**
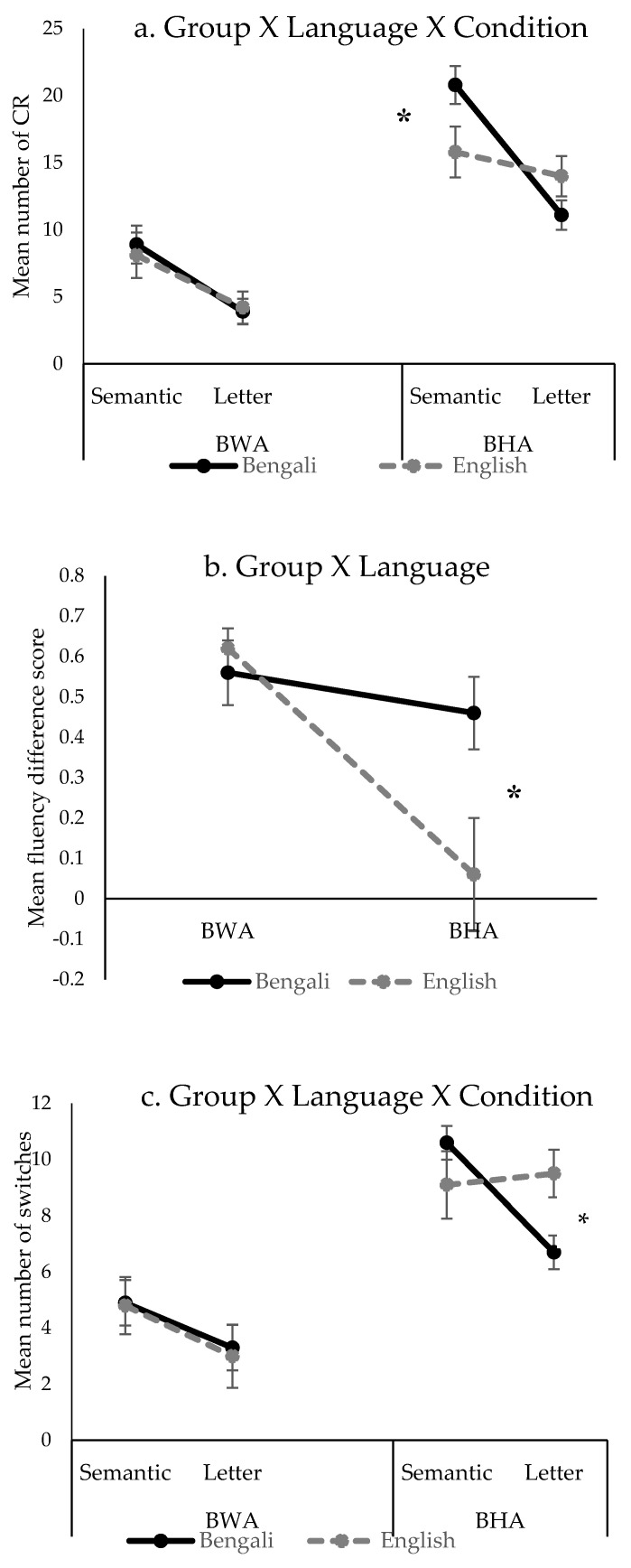
Verbal fluency variables which revealed significant three-way and two-way interactions: a) Mean number of correct responses (CR) (top panel); b) Mean fluency difference score (middle panel); c) Mean number of switches (lower panel). Error bars represent standard error of the means. BWA: Bilinguals with Aphasia: BHC: Bilingual Healthy Controls. *—represents significant difference (p < 0.05).

Differences between BWA and BHC in terms of main effect of Group or an interaction of Group with other factors were observed for: CR, FDS, 1st-RT, switches, and between-cluster pauses. There were no group differences in sub-RT, cluster size, and within-cluster pauses. Figure 1 depicts the significant interactions (Figure 1a, CR: Group X Language X Condition, Figure 1b, FDS: Group X Language, Figure 1c: Number of switches: Group X Language X Condition). 

The CR showed a main effect of Group (BWA: *M* = 6.3, *SD* = 3.2; BHC: *M* = 15.4, *SD* = 3.2) and Condition (Semantic: *M* = 13.4, *SD* = 2.9; Letter: *M* = 8.3, *SD* = 2.2) and a significant three-way interaction of Group X Language X Condition with a large effect size of 0.73 (see Figure 1a). Post hoc analysis of the interaction revealed that there were no significant cross-linguistic differences either for semantic (Bengali: *M* = 8.9, *SD* = 4; English: *M* = 8.1, *SD* = 5.2; *p* = 0.60) or letter (Bengali: *M* = 3.9, *SD* = 2.8; English: *M* = 4.2, *SD* = 3.9; *p* = 0.79) condition for the BWA group. However, the BHC group performed significantly better in Bengali compared to English in semantic fluency (Bengali: M = 20.8, SD = 4; English: *M* = 15.7, *SD* = 5.2; *p* = 0.005). As expected, BWA as a group showed significant word retrieval difficulties compared to BHC in both languages and in both fluency conditions. 

For FDS, there was a main effect of Group (BWA: *M* = 0.59, *SD* = 0.19; BHC: *M* = 0.26, *SD* = 0.24) and a significant two-way interaction of Group X Language with a large effect size of 0.60 (see Figure 1b). Post hoc analysis of the interaction revealed no significant cross-linguistic difference for the BWA group (Bengali: *M* = 0.56, *SD* = 0.19; English: *M* = 0.62, *SD* = 0.34; *p* = 0.59), but the BHC group had a significantly smaller FDS score in English compared to Bengali (Bengali: *M* = 0.46, *SD* = 0.19; English: *M* = 0.06, *SD* = 0.34; *p* = 0.004). Smaller FDS scores for BHC compared to BWA suggests superior executive control abilities for BHC. 

In terms of timing measures, there was a main effect of Group (BWA: *M* = 4.9, *SD* = 3.9; BHC: *M* = 1.3, *SD* = 0.4) for the 1st-RT, but not for the Sub-RT (BWA: *M* = 19.5, *SD* = 4.6; BHC: *M* = 22.5, *SD* = 1.7). Slower 1st-RT suggests BWA took a significantly longer time to initiate the first response, suggesting difficulty in accessing the lexical store at the beginning of the task. However, a comparable Sub-RT indicates that once BWA were able to access the lexical store, they were successful in maintaining the rate of recall throughout the 60 s of the task, and no structural difficulties with the mental lexicon.

For the clustering and switching analysis, there was a main effect of Condition for the cluster size, with a bigger cluster size in semantic fluency compared to letter fluency. Number of switches evidenced a significant main effect of Group (BWA: *M* = 4, *SD* = 2.2; BHC: *M* = 9, *SD* = 1.8), Conditions (Semantic: *M* = 7.4, *SD* = 2.3; Letter: *M* = 5.6, *SD* = 2.2), a significant two-way interaction of Language X Condition with a large effect size of 0.65, and a three-way interaction of Group X Language X Condition with a large effect size of 0.70 (see Figure 1c). Post hoc analysis of the three-way interaction revealed that there was no significant cross-linguistic difference either for semantic or letter condition for the BWA group. However, BHC switched significantly more in English compared to Bengali (Bengali: *M* = 6.7, *SD* = 1.8; English: *M* = 9.5, *SD* = 2.4; *p* = 0.01) in letter fluency. This could mean that the BHC group have used switching as a successful strategy to produce newer exemplars especially in their non-dominant language, English, where there could be greater cross-linguistic interference from the dominant language (i.e., Bengali). For within-cluster pauses, there were no main or interaction effects. For between-cluster pauses, there was only a main effect of Group (BWA: *M* = 9.2, *SD* = 4; BHC: *M* = 4.5, *SD* = 0.64); BWA showed significantly longer between-cluster pauses compared to BHC. Longer between-cluster pause with reduced switching abilities for BWA indicates a difficulty in executive control component (in addition to the difficulty in lexical access) of the verbal fluency task.

### 4.2. Verbal Fluency Performance at the Individual Level.

Table 7 presents the raw scores of each BWA in each condition averaged across languages for the selected verbal fluency variables (number of CR, FDS, cluster size, number of switches). At the individual level, we observed letter fluency to be difficult compared to semantic fluency. For number of CR, compared to BHC, four out of eight BWA were affected on semantic fluency, but seven out of eight BWA showed a significantly lower score on letter fluency. The lower performance on letter fluency was observed in FDS, where seven out of eight BWA performed significantly worse compared to controls. For cluster sizes, BWA performed similarly in two conditions compared to controls. Condition difference was observed for number of switches variable, where three BWA performed significantly poorer compared to controls on the semantic fluency condition, whereas six BWA were affected in the letter fluency condition. Previously in the individual level results (Table 5) section of executive control measures, we observed BWA2, BWA3, BWA6, and BWA8 were most affected compared to BHC. We see the similar pattern here, that is the same participants were most affected across the verbal fluency variables (BWA2: 5 out of 8; BWA3: 5 out of 8; BWA6: 6 out 8; BWA8: 7 out 8). Therefore, individual level results provide further support to the hypothesis that BWA who were affected in the executive control measures, were also affected in a greater proportion in the verbal fluency condition (i.e., letter fluency) where executive control demands were higher. 

### 4.3. Verbal Fluency Performance and Executive Control Measures

**Figure 2:**
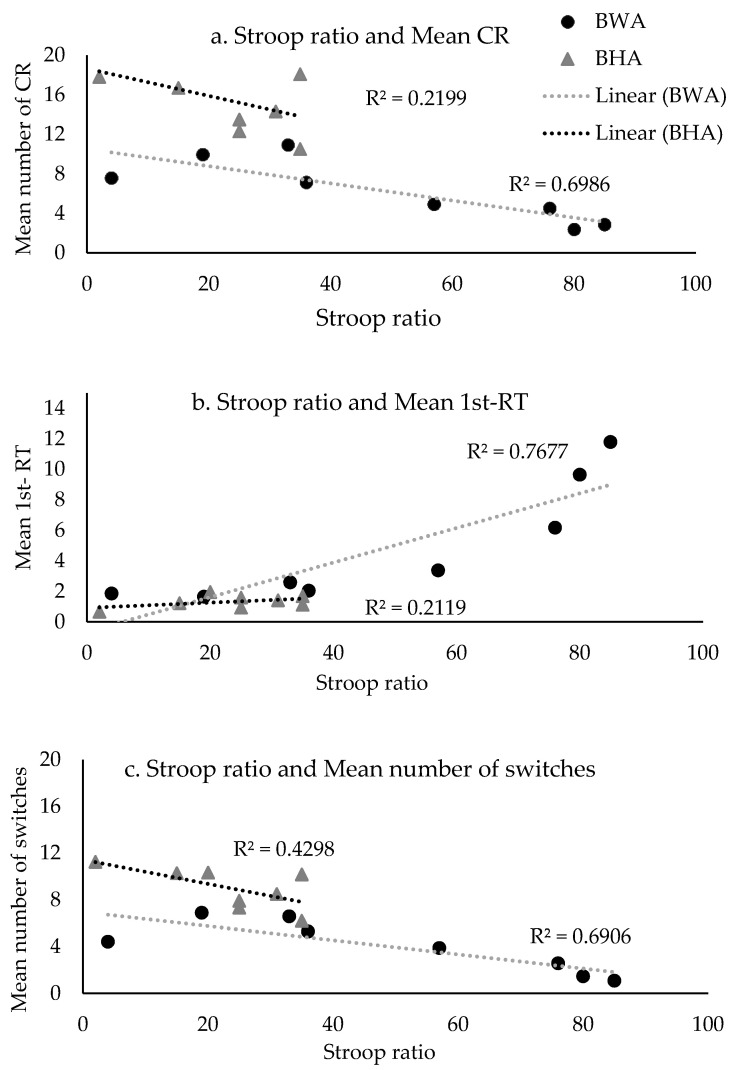
Correlation plots for the significant correlations between the Stroop ratio and verbal fluency parameters: a) Mean number of CR, b) mean 1st-RT, c) mean number of switches for the two groups. BWA: Bilinguals with Aphasia: BHC: Bilingual Healthy Controls.

Figure 2 provides the scatterplots for the significant correlations. BWA showed a significant correlation for the Stroop ratio with CR (negative), 1st RT (positive), and number of switches (negative). BWA with a smaller Stroop ratio (i.e., better inhibitory control) produced a larger number of correct responses, took less time to produce the first response, and switched more between clusters. For the BHC group, the results showed a significant correlation for backward digit span with between-cluster pauses. BHC with a higher backward digit span score (i.e., better working memory) took less time to switch between clusters. As explained in Section 1.1, group size was too small to interpret the null results. Therefore, we do not discuss the null findings further and provide the results in the Table A4 (Appendix A) for future researcher interested in data mining or data analysis. 

## 5. Discussion

In this research, we used verbal fluency tasks to investigate the word production characteristics of Bengali–English BWA to determine the executive control underpinnings for manifestations of their performance, and to identify how the performance is modulated by bilingualism related variables. For the verbal fluency tasks (both semantic and letter conditions), we used a wide range of variables—CR, FDS, 1st-RT, Sub-RT, clustering and switching, within-cluster pause and between-cluster pauses—that are thought to differentially contribute to the lexical and executive components of verbal fluency task. In addition, we measured executive control in the domains of inhibition, switching, and working memory to establish the relationship between executive control and verbal fluency. To summarize the main findings, compared to BHC, BWA showed differences in both lexical and executive control domains as identified in Table 8. 

On the verbal fluency tasks, BWA produced fewer numbers of CR (lexical and executive control), had larger FDS scores (executive control), took longer to access the lexicon (lexical control) at the beginning of the task; switched fewer times (executive control), and took longer time to switch between clusters (executive control). Both groups showed similar clustering scores (lexical), similar retrieval time (lexical), and took similar time to access new words once a subcategory had been accessed, as indicated by within-cluster pauses (lexical). On the separate executive control measures, BWA showed difficulty in the inhibitory control and task switching measures, but were comparable in working memory. On the correlation analysis, BWA showed significant correlations between the executive control measures (inhibitory control) and the verbal fluency measures (number of CR, 1st-RT, and number of switches), whereas BHC showed a significant correlation only for working memory (backward digit span) and between-cluster pauses. On the individual level analysis, BWA who were most affected in the executive control measures also showed greater impairment in the difficult letter fluency condition (BWA2, BWA3, BWA6, and BWA8), compared to BHC. Despite clear cross-linguistic advantage in Bengali for BHC, no cross-linguistic differences were observed in BWA. Overall, the BWA group showed specific differences with respect to lexical retrieval as well as executive control components of the verbal fluency tasks, which was supported by the findings from the separate executive control measures and correlations. 

Compared to BHC, BWA retrieved and generated fewer correct words irrespective of the fluency condition; this corroborates with the aphasia literature, which has shown persons with aphasia to have difficulties in lexical retrieval and production [7,15,16,30]. We performed individual level analysis to further investigate the effect of condition (semantic versus letter) and found several of BWA were affected more in letter fluency compared to semantic fluency. Importantly, findings from the individual analysis in the verbal fluency tasks mirrored to the findings from the individual analysis in the executive control tasks. Specifically, BWAs (BWA 2, 3, 6, 8) who were most affected on the letter fluency condition were also those who had significant difficulties with executive control abilities. This finding supports the notion that BWA found letter fluency difficult, which has been attributed to greater executive control demand [6,19,27,28,30,31,32]. 

Another important finding is that BWA4 did not perform significantly different than the BHC on any of the verbal fluency measures. BWA4 also showed preserved executive control abilities when compared to BHC across all the executive control measures (Stroop ratio, TMT ratio, backward digit span). Therefore, individuals’ findings from the verbal fluency performance are consistent with the performance on executive control measures, which signifies the importance of including separate executive control measures and individual level analysis. 

In terms of cross-linguistic differences, BWA showed no cross-linguistic differences in either conditions; whilst BHC showed better performance in semantic fluency in their dominant and most used language (Bengali). Better performance in Bengali for the semantic fluency task for BHC is consistent with the literature, which assumes current language usage as one of the important factors in verbal fluency performance [15]. At the group level, the lack of cross-linguistic differences in BWA is consistent with the findings in the aphasia literature involving verbal fluency tasks [15,16]. As groups, both groups were Bengali dominant and used Bengali more frequently than English. As a result, both groups were expected to have better performance in Bengali compared to English, but this was borne out only for the BHC group. Comparable or parallel performance in the two languages is the most common observation in BWA [57,58] as well as in other bilingual neurological impairment populations such as bilinguals with Traumatic Brain Injury [11]. Therefore, it is not a surprise that as a group, we see parallel performance in the BWA group in the two languages. For participants (BWA1, BWA4, BWA5, and BWA7) from whom we have WAB performance in both languages, they showed similar severity and type of aphasia in both languages. 

What could the lack of cross-linguistic difference be attributed to? A first potential reason could be a floor effect in the two languages. This is clearly not the case, BWA did not show a floor effect. A second potential reason is that this is an epiphenomenon of their executive control abilities. To perform a fluency task, participants have to access the mental lexicon and retrieve words of the specific language, select words meeting certain constraints, and avoid repetition. It is well-established that successful performance in verbal fluency requires the integrity of both the lexical and executive control processes. The results from this study revealed that BWA showed significant difficulties with the verbal fluency variables that depended more on the executive component of the task (e.g., lower FDS scores, limited number of switching, difficulty in switching) as well as impaired executive control on independent measures (e.g., inhibitory control, mental set shifting). Weakened executive control abilities in BWA may have limited their ability to take advantage of their most used and dominant language and may have diminished the effects of dominance and use. 

A smaller FDS score for BHC in English is suggestive of recruitment of stronger executive control processing in English to overcome the cross-linguistic competition faced from their dominant language (Bengali). Another possible reason for the smaller FDS score in English compared to Bengali can be attributed to the nature of the letter fluency task where BHC performed better in English compared to Bengali. All of our BHC participants were highly educated (except BHC2) and had English as their writing medium. As letter fluency is not a natural way of organizing the mental lexical and depends on the phonology of the language, better education in English might have contributed to a smaller FDS in English. However, the present study did not have any sensitive measure of writing proficiency which could confirm the relationship between writing proficiency in a particular language and performance in the letter fluency. Future studies may consider investigating the role of writing proficiency and its influence on the productivity in letter fluency in a specific language.

On the timing analysis, BWA were significantly slower to initiate the first response as evident by longer 1st RT, suggestive of longer preparation time required. However, there was no significant difference between the two groups on Sub-RT. As explained earlier in the Introduction, shorter Sub-RT with fewer number of CR indicate loss in mental lexicon structure [38]. No differences in Sub-RT is also in line with the results from the background measures (Croft’s material). BWA showed intact verbal semantic comprehension, relatively preserved word repetition and reading aloud abilities. Together, these results provide evidence that BWA in this study showed an intact semantic system with deficits either in the phonological output lexicon or in lexical access (accessing the phonological word form from the semantic system).

On the clustering and switching analyses, compared to BHC, BWA had similar cluster size and within-cluster pause, but fewer number of switches and longer between-cluster pauses. Previous studies have shown reduced cluster size and longer within-cluster pauses in persons with aphasia compared to heathy participants indicating limited lexical resources and/or difficulty in accessing the lexical store and generalized slowing in terms of processing speed [7,15]. In Bose et al.’s study [7], the aphasia group consisted of monolingual speakers (PWA) with a mixture of 11 fluent, 17 non-fluent, and six mixed aphasia. Participants in the Baldo et al.’s study [30] were two native English speakers with moderate severity of aphasia (one fluent and another non-fluent) and the non-fluent PWA showed normal cluster score on the semantic fluency condition, but impaired reduced cluster score on letter fluency. Participants in the Kiran et al.’s study [15], were 10 Spanish–English BWA who were not defined in terms of type and severity of aphasia. However, all their participants showed difficulty in single word comprehension on both languages (English: 47.96%; Spanish: 69.26%). No difference in cluster size and within-cluster pause, but difficulty in switching between one cluster to another can be attributed to the type of aphasia in our BWA group. 

In the present study, all BWA were of non-fluent type. Difficulty in switching with relatively preserved ability to access the mental lexicon is a marker of focal frontal lobe lesions [10]. Therefore, non-fluent BWA may not show difficulty in accessing words within a cluster, and once a cluster was accessed, the retrieval of words within the cluster was not affected. However, BWA showed difficulty in switching between one cluster to another in both semantic and letter fluency conditions, which corroborates with the previous literature involving persons with aphasia [7,15]. Both Bose et al.’s [7] and Kiran et al.’s study [15] found reduced number of switches in the semantic fluency condition for persons with aphasia compared to healthy adults. The present study supports the previous findings, but at the same time extends the literature to show that the difficulty in switching from one cluster to another was evidenced not only in the semantic fluency condition but also in the letter fluency condition. Further, BWA in the present study took a longer time to switch from one cluster to another, which is again supportive of Bose et al.’s finding which showed a reduced number of switches to correlate with longer between-cluster pauses. Reduced number of switches in conjunction with longer between-cluster pauses for BWA is indicative of difficulty with the executive control component of the task [18]. In the present study, intact semantic comprehension on the background language task, no differences in Sub-RT, similar clustering strategy, intact retrieval of words within a cluster with impaired switching, and longer between-cluster pauses for the BWA indicate greater impairment in the executive control components of the verbal fluency task. 

Difficulty in the executive control components of the verbal fluency task for the BWA was further supported by the results obtained from the separate executive control measures. As expected, compared to BHC, BWA showed significantly larger Stroop ratio or difficulty in the inhibitory control component of the Stroop test. The findings are consistent with previous studies on aphasia and inhibitory control [13]. On the task switching measure, BWA showed a larger TMT difference compared to BHC indicative of difficulty in switching between mental sets. Previous studies have shown persons with aphasia to have difficulty in task switching compared to healthy adults [59,60]. We did not observe any difference between the two groups for the backward digit span test, which could be attributed to the difficulty level of this test. Future studies would benefit from using more sensitive experimental measures of working memory (e.g., N-Back task). 

In terms of individual level analysis, not all the BWA showed executive control impairments on all the domains. BWA1, BWA4, BWA5, and BWA7 had relatively preserved executive control abilities across the three domains (inhibitory control: Stroop ratio, mental-set shifting: TMT ratio, and working memory: Backward digit span). These results signify the importance of including a broad range of executive control measures and also the importance of delving at the individual level data for aphasia. 

Finally, the correlation analyses revealed an association between executive control measures and verbal fluency measures. BWA with a smaller Stroop ratio or better inhibitory control produced a larger number of correct responses, took less time to produce the first response, and switched more between clusters. Switching between clusters has been linked to the executive control aspect of the verbal fluency tasks [7] and to be a strong predictor for total CR [19]. The present study confirms the relationship of the executive control, especially of the inhibitory control abilities with the switching component of the verbal fluency task. However, Faroqi-Shah et al. [13] did not find any relationship between the Stroop conflict ratio and the number of correct responses for their BWA. Faroqi-Shah et al. [13] measured the number of CR only for semantic fluency, but not for letter fluency. As previously discussed, executive control demands are higher in the letter fluency condition. Therefore, collapsing across two conditions may have resulted in the significant correlations between Stroop ratio and verbal fluency measures for the present study, signifying the role of executive control abilities during word production in BWA population. We acknowledge that it might be better to investigate the correlation separately for semantic fluency and letter fluency condition. However, given the sample size, we could not address this, and future studies will benefit from having a large sample and investigated the relationship between the two verbal fluency conditions and executive control measures to delineate the role of executive control measures in letter fluency.

## 6. Conclusions

To the best of our knowledge, this is the first study to provide evidence that the lexical and cognitive underpinnings of the verbal fluency performance in BWA can only be explained by having a multi-pronged approach, that is including a broad range of verbal fluency (quantitative, timing measures, clustering and switching; semantic and letter fluency conditions) and independent executive control measures (inhibitory control, mental set-shifting, and working memory). Further, we highlight the importance of analyzing aphasia data at the individual level due to the high variability in this group. Importantly, BWA who were most affected in the executive control measures also showed greater impairment in letter fluency condition. This research makes a significant contribution to our understanding of lexical and executive control aspects in BWA in an under-reported language (i.e., Bengali). Despite being world’s seventh most spoken language, very little is known about the language breakdown following aphasia in this population. In this study, we provide detailed characterizations of the language breakdown in both Bengali and English following stroke and contribute to the literature of aphasia in South-Asian languages. 

## Figures and Tables

**Table 1:** Description of the verbal fluency variables and relative contribution of lexical and executive control processes for each of these variables. Adapted from Patra et al. [6].

Parameters	Description	Lexical Control	Executive Control
**Quantitative analysis**			
Number of correct responses (CR)	Number of words generated in 60-s excluding errors. Measures word retrieval abilities.	√	√
Fluency difference score (FDS) ^1^	Measures the ability to maintain the performance in the demanding condition (i.e., letter fluency).		√
**Time-course analysis** ^2,3^			
1st RT	Time duration from the beginning of the trial to the onset of first response. Measures the preparation time.	√	
Sub-RT	Average of time intervals from the onset of first response to the onset of each subsequent response. Estimate for mean retrieval latency and represents the time point at which half of the total responses have been generated.		√
**Clustering & Switching analysis** ^4^			
Cluster size	Strategic process that helps with generating words within a subcategory and utilizes the speaker’s ability to access words within subcategories.	√	
Number of switches	Strategic process to shift efficiently to a new subcategory when a subcategory is exhausted.		√
Within-cluster pauses	Mean time differences between each successive word within the same cluster.	√	
Between-cluster pauses	Mean time difference between the onset time of the last word of a cluster and first word of the consecutive cluster.		√

^1^ [27]; ^2^ [28]; ^3^ [37]; ^4^ [19].

**Table 2:** Demographic profiles of each BWA, mean and SD of BWA and BHC groups, and the statistical results comparing the groups.

Variables	BWA1	BWA2	BWA3	BWA4	BWA5	BWA6	BWA7	BWA8	BWA		BHC(*N*=8)		Statistical Results
									*M*	*SD*	*M*	*SD*	
Age	50	58	50	54	35	35	34	66	47.4	12.9	44.9	16.5	*t*(14) = 0.67, *p* = 0.51
Sex	M	F	M	M	F	M	M	M	F(2)	M(6)	F(2)	M(6)	χ2(1)1)ele7) = 0.02, p = 0.971ses in ime course analysis, and qualitaitve h each other and whicj measure best predict the n the verbal = 0, *p* = 1
Years of education	18	12	17	18	20	16	16	16	16.6	2.5	16.8	1.8	*t*(14) = −0.23, *p* = 0.82
Time post onset (months)	17	58	19	12	27	40	22	27	27.8	14.8			
Pre-stroke occupation	Accountant	Business	Marketing	General Manager	PhD student	Software Engineer	Marketing	Clerk					
Aphasia type ^1^													
Bengali	Broca’s	Broca’s	CT ^2^	TCM ^3^	Broca’s	CT ^2^	Broca’s	Broca’s					
English	Broca’s	CT ^2^	Broca’s	TCM ^3^	Broca’s	Broca’s	Broca’s	CT ^2^					
Severity^1^													
Bengali	Moderate	Moderate	CT ^2^	Mild	Mild	CT ^2^	Mild	Moderate					
English	Moderate	CT ^2^	Moderate	Mild	Moderate	Severe	Mild	CT ^2^					
AQ ^4^													
Bengali	68.6	75	CT ^2^	83.6	76.8	CT ^2^	77.2	68.6					
English	64.4	CT ^2^	74.4	79.8	74.2	48	76.4	CT ^2^					

Note: ^1^—Type and severity of aphasia were classified based on WAB-R in English and the adapted version in Bengali; ^2^—Could not be Tested due to unavailability. BWA: Bilinguals with Aphasia, BHC: Bilingual Healthy Controls; ^3^—Transcortical Motor; ^4^—AQ was calculated by using the following formula {AQ = (SS score + AVC score + Repetition score + Naming score) × 2}, AQ ratings = Mild (76 and above), Moderate (51–75), Severe (26–50), Very severe (0–25).

**Table 3:** Language background questionnaire scores of each BWA, means and standard deviations of BWA and BHC groups, and the statistical results comparing the groups.

Measures	BWA1		BWA2		BWA3		BWA4		BWA5		BWA6		BWA7		BWA8								
	Pre	Post	Pre	Post	Pre	Post	Pre	Post	Pre	Post	Pre	Post	Pre	Post	Pre	Post	BWA						
																	Pre		Post		BHC (*N* = 8)		
																	*M*	*SD*	*M*	*SD*	*M*	*SD*	Statistical Results ^8^
**Bengali**																							
*LAH ^1,6^*	16		16		12		15		14		12		14		14		14.1	1.5			14.9	1.1	*t*(14) = −1.1, *p* = 0.29
*LOI ^2,6^*	9		6		6		9		9		3		6		8		7	2.1			6.8	2.5	*t*(14) = 0.10, *p* = 0.91
*SLF ^3,6^*	7	5	7	4.5	2.8	2.5	7	5.2	7	4.5	5.5	3.5	6	4.8	7	4.2	6.2	1.5	4.3	0.8	6.6	0.57	*t*(14) = −0.77, *p* = 0.45
*Speaking*	7	4	7	4	4.5	3	7	5	7	4	7	2	7	6	7	3	6.7	0.9	3.9	1.2	7		
*Comprehension*	7	7	7	6	5	5	7	6	7	6	7	6	7	7	7	6	6.7	0.7	6.1	0.6	7		
*Reading*	7	6	7	4	1	1	7	5	7	4	4	3	5	3	7	4	5.6	2.2	3.7	1.5	6.2	1	
*Writing*	7	3	7	4	1	1	7	5	7	4	4	3	5	3	7	4	5.6	2.2	3.4	1.2	6.1	1.3	
*Language use ^4,6^*	30	30	30	30	17	14	30	30	24	26	19	13	23	26	30	30	25.4	5.4	24.8	7.2	25.8	7.7	*t*(14) = −0.11, *p* = 0.91
*LD ^5,7^*	23		26		12		23		25		19		11		26		20.6	6			20.9	5.8	*t*(14) = −0.08, *p* = 0.93
**English**																							
*LAH ^1,6^*	2		0		3		1		5		4		1		0		2	1.8			2.9	1.4	*t*(14) = −0.10, *p* = 0.31
*LOI ^2,6^*	3		0		9		6		2		9		9		3		5.1	3.6			5.6	1.3	*t*(14) = −0.37, *p* = 0.72
*SLF ^3,6^*	6.5	4.4	3.8	2.2	6	4.8	6	4.1	5.6	4	7	4.8	7	4.5	4.2	2.7	5.8	1.2	3.9	.9	4.8	1.5	*t*(14) = 1.3, *p* = 0.21
*Speaking*	6	2	2	2	6	4	6	3.5	4.5	3	7	3	7	3	3	2	5.2	1.9	2.8	.7	4.6	1.8	
*Comprehension*	6	6	3	3	6	6	6	5	6	5	7	6	7	6	4	3	5.6	1.4	5	1.3	4.8	1.8	
*Reading*	7	6	5	2	6	5	6	4	6	4	7	5	7	4	5	3	6.1	0.8	4.1	1.2	5.2	1	
*Writing*	7	3.5	5	2	6	4	6	4	6	4	7	5	7	5	5	3	6.1	0.8	3.8	.9	4.7	1.7	
*Language use ^4,6^*	18	13	8	6	24	21	16	12	16	15	21	24	18	15	12	12	16.6	4.9	14.8	5.9	15.1	7.7	*t*(14) = 0.71, *p* = 0.49
*LD ^5,7^*	7		2		17		9		8		20		23		5		11.4	7.6			12	4.5	*t*(14) = −0.20, *p* = 0.84

Note: ^1^—Language Acquisition History: Maximum score possible 16, greater score in one language means greater immersion into that language during childhood; ^2^—Language of Instruction: Maximum score possible 9, greater score in one language means greater number of years of education in that language; ^3^—Self-Language Proficiency: On a scale of zero to seven (0 = no proficiency, ^7^ = native like proficiency), greater score in language means greater proficiency in that language; ^4^—maximum score possible 30, greater score in one language means greater use of that language in daily life; ^5^—Language Dominance: Maximum score possible 31, dominant language is the language which obtains a greater score than the other language; ^6^—adapted from Muñoz et al. [49]; ^7^—language dominance questionnaire [50]. ^8^— independent sample t-test was used to compare pre-stroke ratings of BWA with BHC; BWA: Bilinguals with Aphasia, BHC: Bilingual Healthy Controls.

**Table 4:** Means, standard deviations, and the statistical results of the executive control measures by group (BWA; BHC).

Measures	BWA (*N* = 8)			BHC (*N* = 8)			
	*M*	*Min–Max*	*SD*	*M*	*Min–Max*	*SD*	
Stroop difference	1636	66–4069	1529	200	15–335	113	***U*^1^ = 10, *p* = 0.02**
Stroop ratio	49	4–85	30	24	3–35	11	***t*(8.9) = 2.2, *p* = 0.05**
TMT difference	193	33–759	246	32	11–61	21	***U*** **^1^** **= 13, *p* = 0.005**
TMT ratio	4	1.6–8	2	2	1–3	0.6	***U*** **^1^** **= 13, *p* = 0.05**
Backward digit span	4	3–5	0.8	4.5	3–7	2	*U*^1^ = 27, *p* = 0.64

^1^—Mann-Whitney U test. BWA: Bilinguals with Aphasia, BHC: Bilingual Healthy Controls. TMT: Trail Making Test. Shaded bold texts represent significant differences (*p* < 0.05).

**Table 5:** Statistical results of single case analysis to compare an individual’s (BWA) test score against control (BHC) for each executive control measure.

	Inhibitory control		Mental-set shifting		Working memory
BWA	Stroop difference	Stroop ratio	TMT difference	TMT ratio	Backward digit span
BWA1	**1132.8**	36	66	2.8	4
BWA2	**4068.8**	**76**	**316**	**8**	3
BWA3	**1296.1**	**57**	**82**	1.9	3
BWA4	367.4	33	32.6	1.6	4
BWA5	65.7	4	**123**	2.8	4
BWA6	**3511.7**	**80**	**129**	2.8	5
BWA7	251.8	19	37.1	2.7	5
BWA8	**2391.3**	**85**	**759**	**6.7**	3
BHC (*Mean*, *SD*)	199.8, 113.4	24, 11.1	31.8, 20.7	2.0, 0.6	4.5, 1.6

Note—Crawford and Howell’s [53] statistical method was used to compare each BWA’s score with the BHC group. Singlism.exe program [54] was used to compute the statistics, and the shaded bold texts represent significant *p*-values (*p* < 0.05), where BWA’s score was significantly different than the BHC group mean.

**Table 6:** Means, standard deviations, and the summary of statistical results of the dependent variables by Group, Conditions, and Language.

Measures	BWA (N = 8)						BHC (N = 8)						Statistical Analysis (Group, Language, Condition)						
	B		E		Total		B		E		Total								
	*M*	*SD*	*M*	*SD*	*M*	*SD*	*M*	*SD*	*M*	*SD*	*M*	*SD*	Group (G)	Lang (L)	Cond (C)	G x L	G x C	C x L	G x L x C
CR ^1^	6.4	3.1	6.1	3.7	6.3	3.1	16.0	3	14.9	4.4	15.4	1.2	Sig	NS	Sig	NS	NS	Sig	NS
*Semantic*	8.9	4	8.1	4.8	8.5	3.9	20.8	4	15.8	5.6	18.3	4.3							
*Letter*	3.9	2.7	4.2	3.4	4.1	2.9	11.1	3	14	4.4	12.5	3.2							
FDS ^2^	0.56	0.24	0.62	0.26	0.59	0.19	0.46	0.14	0.06	0.40	0.26	0.24	Sig	NS	NA	Sig	NA	NA	NA
1st RT	5.2	5.5	4.6	4.7	4.9	3.9	1.2	0.7	1.5	0.6	1.3	0.4	Sig	NS	NS	NS	NS	NS	NS
*Semantic*	3.4	2.4	5.9	8	4.7	4.4	1.2	0.7	1.2	0.5	1.2	0.3							
*Letter*	6.9	11	3.3	2	5.1	5.8	1.1	0.9	1.8	0.8	1.4	0.5							
Sub-RT	20.4	6.5	18.5	7.3	19.5	4.6	22	2.3	23	1.5	22.5	1.7	NS	NS	NS	NS	NS	NS	NS
*Semantic*	21.2	7.6	16.4	7.6	18.8	5.8	20	3.2	21	3.5	20.6	2.8							
*Letter*	19.6	8.2	20.7	9.7	20.2	5.2	25	2.6	24	3.5	24.5	2.4							
Cluster size	0.49	0.30	0.50	0.23	0.50	0.20	0.74	0.20	0.56	0.12	0.65	0.14	NS	NS	Sig	NS	NS	NS	NS
*Semantic*	0.83	0.53	0.76	0.48	0.79	0.45	0.93	0.24	0.65	0.23	0.79	0.21							
*Letter*	0.16	0.22	0.24	0.21	0.20	0.12	0.55	0.28	0.47	0.21	0.51	0.17							
Switches	4.1	2.1	3.9	2.6	4	2.2	8.7	1.4	9.3	2.5	9	1.8	Sig	NS	Sig	NS	NS	Sig	Sig
*Semantic*	4.9	2.3	4.8	2.9	4.9	2.4	10.6	1.7	9.1	3.3	9.9	2.3							
*Letter*	3.3	2.3	3	3.2	3.2	2.5	6.7	1.8	9.5	2.4	8.1	1.7							
WCP ^3^	3	1.8	3	1.8	3	1.3	3.1	1.1	3.6	2.1	3.4	1.6	NS	NS	NS	NS	NS	NS	NS
*Semantic*	3.8	1.4	3	0.8	3.4	0.65	1.7	0.54	2.3	0.90	2	0.51							
*Letter*	2.2	3.1	3.1	3.2	2.6	2.6	4.4	2.2	5	3.6	4.7	2.8							
BCP ^4^	8.1	2.5	10.3	5.8	9.2	4	4.3	0.78	4.7	0.97	4.5	0.64	Sig	NS	NS	NS	NS	NS	NS
*Semantic*	8.2	5.6	10.3	7.8	9.3	6.4	3.4	0.49	4.6	1.4	4.1	0.75							
*Letter*	7.9	4	10.4	9	9.1	5.2	5.3	1.5	4.7	1.1	5	0.99							

^1^—Number of correct responses, ^2^—Fluency Difference Score, ^3^—Within-Cluster Pauses, ^4^—Between-Cluster Pauses, Condition (Semantic, Letter); BWA: Bilinguals with Aphasia, BHC: Bilingual Healthy Controls; B: Bengali, E: English. Sig represent significant difference (*p* < 0.05), NS represents no significant difference, NA represents not applicable. Shaded bold texts represent significant difference (*p* < 0.05).

**Table 7:** Raw score of each BWA in semantic and letter fluency condition (averaged across languages) for number of correct responses (CR), fluency difference score (FDS), cluster size, and number of switches, and statistical results for each BWA when compared to controls (BHC).

	CR		FDS	Cluster size		Number of switches	
	Semantic	Letter		Semantic	Letter	Semantic	Letter
BWA1	10.75	**3.5**	**0.69**	0.4	0.19	7.75	**2.83**
BWA2	**4.25**	**4.79**	**0.59**	0.72	0.29	**3**	**2.17**
BWA3	**7.5**	**2.34**	**0.68**	**0.27**	0.28	6.25	**1.5**
BWA4	12	9.83	0.19	1.03	0.32	5.5	7.67
BWA5	11	**4.17**	**0.62**	0.9	0.31	6	**2.83**
BWA6	**3.25**	**1.5**	**0.55**	0.61	**0.09**	**1.75**	**1.17**
BWA7	13.75	**6.17**	**0.55**	0.72	**0.14**	7.25	6.5
BWA8	**5.25**	**0.5**	**0.87**	**1.71**	0.35	**1.5**	**0.67**
BHC *(Mean, SD)*	18.3, 4.3	12.5, 3.2	0.26, 0.24	0.79, 0.21	0.51, 0.17	9.9, 2.3	8.1, 1.7

Note—Crawford and Howell’s [53] statistical method was used to compare each BWA’s score with the BHC group. Singlism.exe program [54] was used to compute the statistics and the shaded bold texts represent significant difference (*p* < 0.05), where BWA’s score was significantly different than the BHC group mean.

**Table 8:** Results of the current study in the context of the verbal fluency variables and their lexical and executive control components. The bold fonts indicate significant effects.

Parameters	Processes		Bilinguals with Aphasia (BWA) vs. Bilingual Healthy Controls (BHC)	
	Lexical	Executive	Findings	Correlation with Executive Control
Quantitative analysis				
Number of correct responses	√	√	Yes BWA < BHC	Yes, (negative) with Stroop ratio BWA
Fluency difference score		√	Yes BWA > BHC	No
Time-course analysis				
1st RT	√		Yes BWA > BHC	Yes, (positive) with Stroop ratio for BWA
Sub-RT		√	No BWA = BHC	No
Clustering and Switching analysis				
Cluster size	√		No BWA = BHC	No
Number of switches		√	Yes BWA < BHC	Yes, (negative) with Stroop ratio for BWA
Within-cluster pauses	√		No BWA = BHC	No
Between-cluster pauses		√	Yes BWA > BHC	Yes, (negative) with backward digit span for BHC

Yes—significant findings, No—not significant findings.

## Appendix A

**Table A1:** Western aphasia battery test scores in Bengali (Keshri et al., 2013) and English (Kertesz, 2006) of each BWA.

Subtests of WAB	BWA1		BWA2		BWA3		BWA4		BWA5		BWA6		BWA7		BWA8	
	B	E	B	E	B	E	B	E	B	E	B	E	B	E	B	E
Spontaneous speech																
Information content ^1^	7	7	8	CT ^19^	CT ^19^	9	8	8	8	8	CT ^19^	4	8	8	7	CT ^19^
Fluency ^2^	4	4	4			4	5	4	4	4		2	4	4	4	
Score ^3^	11	11	12			13	13	12	12	12		6	12	12	11	
Auditory verbal comprehension																
Yes/No questions ^4^	60	54	60	CT ^19^	CT ^19^	60	60	60	60	60	CT ^19^	54	60	60	60	CT ^19^
Auditory word recognition ^5^	60	60	60			60	60	60	60	60		56	60	60	60	
Sequential commands ^6^	80	80	80			80	80	80	80	80		66	80	80	80	
Total ^7^	200	194	200			200	200	200	200	200		176	200	200	200	
Score ^8^	10	9.7	10			10	10	10	10	10		8.8	10	10	10	
Repetition																
Repetition ^9^	64	49	65	CT ^19^	CT ^19^	77	100	90	78	76	CT ^19^	30	78	76	65	CT ^19^
Score ^10^	6.4	4.9	6.5			7.7	10	9	7.8	7.6		3	7.8	7.6	6.5	
Naming																
Object naming ^11^	42	38	54	CT ^19^	CT ^19^	42	57	54	57	48	CT ^19^	45	57	57	45	CT ^19^
Fluency ^12^	10	16	7			11	12	16	10	11		5	12	13	8	
Sentence completion ^13^	9	8	6			8	9	9	9	8		6	9	8	8	
Responsive speech ^14^	8	4	8			4	10	10	10	8		6	10	8	8	
Total ^15^	69	66	75			65	88	89	86	75		62	88	86	69	
Score ^16^	6.9	6.6	7.5			6.5	8.8	8.9	8.6	7.5		6.2	8.8	8.6	6.9	
AQ ^17^	68.6	64.4	75			74.4	83.6	79.8	76.8	74.2		48	77.2	76.4	68.6	
Type ^18^	BA	BA	BA			BA	TCM	TCM	BA	BA		BA	BA	BA	BA	

Note: ^1^—maximum possible score 10; ^2^—maximum possible score 10; ^3^—sum of information content and fluency score; ^4^—maximum possible score 60; ^5^—maximum possible score 60, ^6^—maximum possible score 80; ^7^—sum of yes/no question, auditory word recognition and sequential commands; ^8^—total score divided by 20; ^9^—maximum possible score 100; ^10^—repetition score divided by 10; ^11^—maximum possible score 60; ^12^—maximum possible score 20; ^13^—maximum possible score 10; ^14^—maximum possible score 10; ^15^—sum of all the naming subtests scores; ^16^—total divided by 10; ^17^—AQ was calculated by using the following formula {AQ = (SS score + AVC score + Repetition score + Naming score) × 2} and severity can be classified as Mild (76 and above), Moderate (51–75), Severe (26–50), Very severe (0–25); ^18^—Aphasia type: BA represents Broca’s aphasia and TCM represents Transcortical Motor aphasia; ^19^—Could not be Tested due to unavailability. BWA: Bilinguals with Aphasia; B: Bengali, E: English.

**Table A2:** Accuracy (raw score and %) in language background test of BWA using Croft’s Test Battery.

	BWA1	BWA2	BWA3	BWA4	BWA5	BWA6	BWA7	BWA8	BWA (*N* = 8)		BHC (*N* = 8)		
									M	SD	M	SD	Statistical Analysis ^4^
Croft’s test battery ^1^													
Naming ^2^													
*Bengali*	28(93.3%)	23(76.7%)	15(50%)	29(96.7%)	29(96.7%)	10(33.3%)	29(96.7%)	22(73.3%)	23.1(77%)	7.2(24.1%)	30(100%)		***t*(7) = 2.7, *p* = 0.03**
*English*	22(73.3%)	2(6.7%)	26(86.7%)	29(96.7%)	24(80%)	23(76.7%)	29(96.7%)	15(50%)	21.1(70%)	8.9(29.8%)	30(100%)		***t*(7) = 2.7, *p* = 0.03**
*Difference ^3^*	*p* = 0.39	***p* < 0.001**	*p* = 0.08	*p* = 1	*p* = 0.49	***p* = 0.02**	*p* = *1*	*p* = 0.24	*p* = 0.63		*p* = 1		
Repetition ^2^													
*Bengali*	21(70%)	27(90%)	22(73.3%)	30(100%)	27(90%)	20(66.7%)	30(100%)	30(100%)	25.9(86.3%)	4.3(14.1%)	30(100%)		***t*(7) = 2.7, *p* = 0.03**
*English*	22(73.3%)	CNP^5^	26(86.7%)	28(93.3%)	30(100%)	24(80%)	30(100%)	30(100%)	27.1(90.5%)	3.2(10.8%)	30(100%)		*t*(6) = 2.3, *p*= 0.06
*Difference ^3^*	*p* = 0.88		*p* = 0.56	*p* = 0.79	*p* = 0.69	*p* = 0.54	*p* = 1	*p* = 1	*p* = 0.15		*p* = 1		
Word to picture matching ^2^													
*Bengali*	30(100%)	30 (100%)	30(100%)	30(100%)	30(100%)	30(100%)	30(100%)	30(100%)	30(100%)		30(100%)		*p* = 1
*English*	30(100%)	30(100%)	30(100%)	30(100%)	30(100%)	30(100%)	30(100%)	30(100%)	30(100%)		30(100%)		*p* = 1
*Difference ^3^*	*p* = 1	*P* = 1	*p* = 1	*p* = 1	*p* = 1	*p* = 1	*p* = 1	*p* = 1	*p* = 1		*p* = 1		
Reading Aloud ^2^													
*Bengali*	23(76.7%)	16(53.3%)	CNP^5^	29(96.7%)	28(93.3%)	12(40%)	24(80%)	30(100%)	23.1(87.1%)	6.8(21.9%)	30(100%)		***t*(6) = 2.6, *p* = 0.04**
*English*	19(63.3%)	CNP^5^	27(90%)	29(96.7%)	29(96.7%)	24(80%)	30(100%)	CNP^5^	26.3(87.8%)	4.2(13.9%)	30(100%)		*t*(5) = 2.1, *p* = 0.08
*Difference ^3^*	*p* = 0.53			*p* = 1	*p* = 0.89	***p* = 0.04**	*p* = 0.41		*p* = 0.18		*p* = 1		

^1^—Croft’s test battery [48]; ^2^—Maximum score possible is 30; ^3^—Chi-square test was conducted to compare the cross-linguistic differences for each BWA; ^4^—Independent sample t-test was conducted to compare the differences at the group level; ^5^—Could Not Perform. Shaded bold texts represent significant difference (*p* < 0.05). BWA: Bilinguals with Aphasia, BHC: Bilingual Healthy Controls.

**Table A3:** Statistical results of the verbal fluency variables by Group, Conditions, and Language.

Measures	Statistical Analysis (Group, Language, Condition)						
	Group (G)	Lang (L)	Cond (C)	G x L	G x C	C x L	G x L x C
CR ^1^	**F(1,14) = 32.2, *p* < 0.001,** $\eta_{p}^{2}$ **= 0.70**	F(1,14) = 0.73, *p* = 0.41, $\eta_{p}^{2}$ = 0.05	**F(1,14) = 35.5, *p* = 0.009,** $\eta_{p}^{2}$ **= 0.72**	F(1,14) = 0.27, *p* = 0.61, $\eta_{p}^{2}$ = 0.02	F(1,14) = 0.65, *p* = 0.43, $\eta_{p}^{2}$ = 0.04	**F(1,14) = 27.1, *p* < 0.001,** $\eta_{p}^{2}$ **= 0.66**	**F(1,14) = 15.5, *p* < 0.001,** $\eta_{p}^{2}$ **= 0.53**
*Semantic*							
*Letter*							
FDS ^2^	**F(1,14) = 8.9, *p* = 0.01,** $\eta_{p}^{2}$ **= 0.39**	F(1,14 ) = 4.2, *p* = 0.06, $\eta_{p}^{2}$ = 0.23	NA	**F(1,14) = 8, *p* = 0.01,** $\eta_{p}^{2}$ **= 0.36**	NA	NA	NA
1st RT	**F(1,14) = 6.54, *p* = 0.02,** $\eta_{p}^{2}$ **=** **0.32**	F(1,14) = 0.01, *p* = 0.92, $\eta_{p}^{2}$ = 0.001	F(1,14) = 0.07, *p* = 0.79, $\eta_{p}^{2}$ = 0.005	F(1,14) = 0.14, *p* = 0.72, $\eta_{p}^{2}$ = 0.01	F(1,14) = 0.01, *p* = 0.94, $\eta_{p}^{2}$ = 0.001	F(1,14) = 1.3, *p* = 0.28, $\eta_{p}^{2}$ = 0.08	F(1,14) = 2.1, *p* = 0.17, $\eta_{p}^{2}$ **=** 0.13
*Semantic*							
*Letter*							
Sub-RT	F(1,14) = 3.2, *p* = 0.10, $\eta_{p}^{2}$ = 0.18	F(1,14) = 0.21, *p* = 0.65, $\eta_{p}^{2}$ = 0.01	F(1,14) = 4.1, *p* = 0.06, $\eta_{p}^{2}$ = 0.22	F(1,14) = 0.28, *p* = 0.60, $\eta_{p}^{2}$ = 0.02	F(1,14) = 0.88, *p* = 0.36, $\eta_{p}^{2}$ = 0.06	F(1,14) = 0.62, *p* = 0.44, $\eta_{p}^{2}$ = 0.04	F(1,14) = 2, *p* = 0.17, $\eta_{p}^{2}$ = 0.13
*Semantic*							
*Letter*							
Cluster size	F(1,14) = 3.1, *p* = 0.10, $\eta_{p}^{2}$ = 0.18	F(1,14) = 1.5, *p* = 0.23, $\eta_{p}^{2}$ = 0.10	**F(1,14) = 18.7, *p* < 0.001,** $\eta_{p}^{2}$ **= 0.57**	F(1,14) = 1.7, *p* = 0.21, $\eta_{p}^{2}$ = 0.11	F(1,14) = 2.3, *p* = 0.15, $\eta_{p}^{2}$ = 0.14	F(1,14) = 2, *p* = 0.18, $\eta_{p}^{2}$ = 0.12	F(1,14) = 0.08, *p* = 0.79, $\eta_{p}^{2}$ = 0.005
*Semantic*							
*Letter*							
Switches	**F(1,14) = 24.9, *p* < 0.001,** $\eta_{p}^{2}$ **= 0.64**	F(1,14) = 0.27, *p* = 0.61, $\eta_{p}^{2}$ **=** 0.02	**F(1,14) = 9.7, *p* = 0.008,** $\eta_{p}^{2}$ **= 0.41**	F(1,14) = 0.61, *p* = 0.45, $\eta_{p}^{2}$ = 0.04	F(1,14) = 0.01, *p* = 0.97, $\eta_{p}^{2}$ = 0.000	**F(1,14) = 10.6, *p* = 0.006,** $\eta_{p}^{2}$ **= 0.43**	**F(1,14) = 13.4, *p* = 0.003,** $\eta_{p}^{2}$ **= 0.49**
*Semantic*							
*Letter*							
WCP ^3^	F(1,14) = 0.23, *p* = 0.64, $\eta_{p}^{2}$ = 0.02	F(1,14) = 0.34, *p* = 0.57, $\eta_{p}^{2}$ = 0.02	F(1,14) = 2, *p* = 0.17, $\eta_{p}^{2}$ = 0.13	F(1,14) = 0.27, *p* = 0.61, $\eta_{p}^{2}$ = 0.02	F(1,14) = 6.5, *p* = 0.02, $\eta_{p}^{2}$ = 0.32	F(1,14) = 3.9, *p* = 0.07, $\eta_{p}^{2}$ = 0.22	F(1,14) = 3.6, *p* = 0.08, $\eta_{p}^{2}$ = 0.21
*Semantic*							
*Letter*							
BCP ^4^	**F(1,14) = 10.9, *p* = 0.005,** $\eta_{p}^{2}$ **= 0.44**	F(1,14) = 2.8, *p* = 0.79, $\eta_{p}^{2}$ = 0.17	F(1,14) = 0.08, *p* = 0.79, $\eta_{p}^{2}$ = 0.005	F(1,14) = 1.6, *p* = 0.22, $\eta_{p}^{2}$ = 0.10	F(1,14) = 0.14, *p* = 0.71, $\eta_{p}^{2}$ = 0.01	F(1,14) = 0.16, *p* = 0.69, $\eta_{p}^{2}$ = 0.01	F(1,14) = 0.33, *p* = 0.58, $\eta_{p}^{2}$ = 0.02
*Semantic*							
*Letter*							

Shaded bold texts represent significant statistical results (*p* < 0.05). NA represents not applicable.

**Table A4:** Correlation coefficients between the executive control measures and the verbal fluency variables for each group.

		CR	FDS	1st RT	Sub-RT	Cluster Size	Number ofSwitches	WCP	BCP
BWA (*N* = 8)									
Stroop ratio	rs ^1^	**−0.88**	0.35	**0.95**	−0.52	0.02	**−0.86**	−0.71	0.55
	*p*	**0.004**	0.40	**<0.001**	0.18	0.95	**0.007**	0.05	0.16
TMT ratio	rs ^1^	−0.76	0.39	0.57	−0.67	0.17	−0.71	−0.83	−0.79
	*p*	0.03	0.33	0.14	0.07	0.69	0.05	0.01	0.02
Backward digit span	rs ^1^	0.28	−0.60	−0.48	−0.09	−0.16	0.48	0.25	−0.13
	*p*	0.51	0.12	0.23	0.83	0.70	0.23	0.55	0.77
	BHC (*N* = 8)								
Stroop ratio	rs ^1^	−0.37	0.58	0.46	−0.25	0.48	−0.71	0.67	0.71
	*p*	0.41	0.13	0.25	0.55	0.23	0.05	0.07	0.05
TMT ratio	rs ^1^	−0.33	0.62	0.19	−0.45	0.31	−0.62	0.31	0.36
	*p*	0.42	0.10	0.65	0.26	0.45	0.10	0.46	0.38
Backward digit span	rs ^1^	0.77	0.00	−0.08	0.77	0.23	0.69	−0.62	**−0.85**
	*p*	0.02	1.00	0.89	0.02	0.58	0.06	0.10	**0.008**

^1^—Spearman’s correlation. BWA: Bilingual with Aphasia: BHC: Bilingual Healthy Controls. Shaded bold texts represent significant correlation (*p* < 0.01).
